# A survey of abnormalities in the colon and rectum in patients with haemorrhoids

**DOI:** 10.1186/1471-230X-10-74

**Published:** 2010-07-07

**Authors:** Mark V Koning, Ruud JLF Loffeld

**Affiliations:** 1 Department of Internal Medicine, Zaans Medisch Centrum Zaandam, The Netherlands

## Abstract

**Background:**

Haemorrhoids are a common problem in daily practice. However, symptoms may also be caused by other abnormalities in the rectum or colon. Data on the presence of these abnormalities in patients with haemorrhoids is sparse. To examine the prevalence of abnormalities of the colon or rectum in patients with and without haemorrhoids, stratified for age.

**Methods:**

In a 17-year period 1910 consecutive patients with haemorrhoids and 7936 patients without haemorrhoids were analysed retrospectively. All of these patients had an endoscopic examination for different clinical reasons. All significant endoscopic co-findings (diverticuli, polyps, cancer, angiodysplasia and varices, or colitis) were recorded.

**Results:**

The patients were divided in 2 groups. Group 1 (n = 861 (45.1%)) consisted of patients with only haemorrhoids, group 2 (n = 1049 (54.9%)) consisted of patients with haemorrhoids and another endoscopic diagnosis. Patients in group 1 were significantly younger, mean age 55.3 ± 14.1 years versus 67.4 ± 12.1 years (p < 0.001), and underwent significantly more often a sigmoidoscopy, 11% versus 2% (p < 0.0001). Furthermore, endoscopic co-findings were found with increasing age. The majority of diverticuli, polyps, cancer and vascular lesions were detected in the age group above 50 years, while only colitis was more often present in the younger group. There was no significant difference in gender when group 1 and 2 were compared with the reference group. Diverticuli and angiodysplasia/varices occurred significantly more often in group 2. The other significant diagnoses were diagnosed more often in the reference group.

**Conclusion:**

In patients with haemorrhoids other abnormalities can be present. Especially in older patients the clinician must be cautious to attribute complaints solely to haemorrhoids.

## Background

Haemorrhoids are a common problem in daily practice. The prevalence in the population is approximately 4 percent [1]. Many patients complain of itching and bleeding, and sometimes faecal soiling. Often haemorrhoids are present. Patients recognise the complaints and especially in cases of rectal bleeding they blame the haemorrhoids. However, many other abnormalities in the rectum and colon can be responsible for rectal bleeding or complaints in general. Especially as people get older, there is an increasing possibility that, for instance, adenomas or even cancer is present [2].

Data on abnormalities in the colon and rectum in patients presenting with haemorrhoids is sparse. For this reason a large cross-sectional study was done in order to assess the prevalence of abnormalities in the colon and rectum in patients with haemorrhoids. Furthermore, the results were stratified for age to see in what age category a full endoscopy is mandatory.

## Methods

All consecutive endoscopies from the colon and rectum done in a period of seventeen years at the endoscopy department of the Zaans Medical Centre, the regional hospital for the Zaanstreek region in the Netherlands, were included. The majority of patients (>90%) was scheduled for colonoscopy. The reasons for doing the endoscopy were the normal clinical indications in daily practice (abdominal complaints, diarrhoea, rectal bleeding, anaemia, changes in bowel habits, family history of colorectal cancer and screening), not specifically linked to haemorrhoids.

All patients in whom haemorrhoids were seen were included in the study. Only the first endoscopy, done in a patient was included. Exclusion criterion was endoscopy done because of follow-up after prior diagnosis. Hence, patients developing haemorrhoids while in follow-up were excluded. A procedure was considered as follow-up if it was done within six years after prior endoscopy in which adenomas, cancer or inflammatory bowel disease was diagnosed (maximal normal elapsing time in guidelines for follow-up after removal of adenomas). Prior to the procedure the peri-anal region is inspected. During retrieval of the endoscope the anal canal is inspected as well for the presence of haemorrhoids.

As a reference group all patients without haemorrhoids, in the same study period, was used. In this group all patients in whom no abnormalities were seen in the rectum and colon during endoscopy, as well as patients undergoing regular follow-up because of removed adenomas in the past, cancer or a family history of cancer, were excluded.

All patients underwent endoscopy after standard bowel cleansing. In the beginning of the nineties PEG solution via nasogastric tube was applied, later on ambulatory PEG-solution (Klean Prep^® ^and Moviprep^®^) was used.

The results of endoscopy were noted in a standardised endoscopy report. From 1992 till 2003 handwritten, from 2003 using the Olympus Endobase™ computerised system.

From all patients gender, the age at the time of colonoscopy and the results of colonoscopy were noted. Furthermore, it was recorded whether the colonoscopy was successful in the sense that caecal intubation was achieved.

Significant endoscopic diagnoses were defined as presence of diverticuli, polyps (adenomatous, inflammatory, hyperplastic etc.), cancer, angiodysplasia, and colon or rectum varices.

For statistical analysis the patients were divided in two groups, one with only haemorrhoids (group 1), the other with haemorrhoids and a co-finding (group 2). The Fishers exact or Chi-square testing was used to test frequencies between categorical data. Mann and Whitney U-test was used to check for significant differences in continuous data. All testing was 2-tailed and significant P-values were <0.05. Data are presented as mean ± Standard Deviation (SD). All analyses were done with SPSS version 16.0.

The study was approved by the ethical committee of the Zaans Medical Center.

## Results

In the study period 1910 patients fulfilled the inclusion criterion. Colonoscopy was scheduled in 1777 (93%) patients and sigmoidoscopy in 133. Mean age for patients undergoing colonoscopy or sigmoidoscopy was 62.2 years (SD 14.2) and 57.8 (SD 15.9) respectively (p = 0.001).

Group 1 consisted of 861 patients (45.1%, 409 men, 452 women, mean age 55.3, SD 14.1, range 21-89). These patients underwent 764 (89%) colonoscopies and 97 sigmoidoscopies. Caecal intubation was successful in 84.4% of the cases.

Group 2 consisted of 1049 patients (54.9%, 501 men, 548 women, mean age 67.4, SD 12.1, range 27-96). These patients underwent 1013 (97%) colonoscopies and 36 sigmoidoscopies. Caecal intubation was successful in 88% of the cases.

Patients of group 1 were statistically significant younger than patients from group 2 (p < 0.001), and underwent significantly less often colonoscopy, 89% versus 97% (p < 0.001).

Table 1 shows the outcome of sigmoidoscopy or colonoscopy in patients of group 2. Only polyps were significantly more often found during colonoscopy.

**Table 1:** the outcome of colonoscopy or sigmoidoscopy in patients of group 2.

	Colonoscopy	Sigmoidoscopy	p
N	1013	36	

Diverticuli	724 (71.5%)	31 (86.1%)	0.059

Polyps	361 (35.6%)	5 (13.9%)	0.007

Inflammation	68 (6.7%)	1 (2.8%)	0.507

Carcinoma	48 (4.7%)	0 (0%)	0.405

Angiodysplasia/varices	36 (3.6%)	1 (2.8%)	1.000

**Figure 1:**
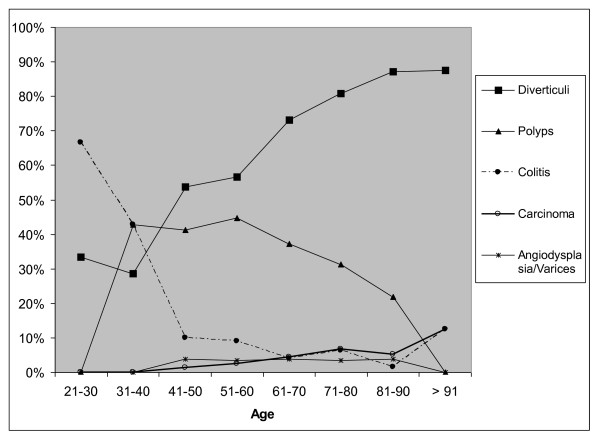
**Diagnoses in patients of group 2 related to the age cohorts. The percentage of positives per age cohort is noted**.

Table 2 shows the diagnoses in group 2 for each age cohort. More than one endoscopic finding can be present in a patient. Significant endoscopic diagnoses were more often seen in patients above the age of 50 years. Figure 1 shows these data graphically.

**Table 2:** different endoscopic diagnoses in patients of different age cohorts.

Number	age	diverticuli	polyps	colitis	cancer	angiodsplasia/varices
3	21-30	1	-	2	-	-

14	31-40	4	6	6	-	-

80	41-50	43	33	8	1	3

201	51-60	114	90	18	5	7

293	61-70	214	109	12	13	11

317	71-80	256	99	20	21	11

133	81-90	116	29	2	7	5

8	>91	7	-	1	1	-

total	755	366	69	48	37	

						

Number	age	diverticuli	polyps	colitis	cancer	angiodsplasia/varices

						

97	<50	48(6)	39(11)	16(23)	1(2)	3(8)

952	≥50	707(94)	327(89)	53(77)	47(98)	34(92)

total	755	366	69	48	37	

More diagnoses per patient are possible (number between brackets denotes the percentage).

p < 0.0001

The reference group consisted of 7936 patients (3547 men (44.7%), 4389 women (55.3%)). There was no significant difference in gender when group 1 and 2 were compared with this reference group. Table 3 shows the yield of endoscopy when group 2 was compared with this reference group. Diverticuli and angiodysplasia/varices occurred significantly more often in group 2. The other significant diagnoses were diagnosed more often in the reference group.

**Table 3:** outcome of endoscopy in group 2 and the reference group (number between brackets is percentage).

	reference group	group 2	p-value
number	7936	1049	

Diverticuli	3790 (47.7)	755 (71.9)	<0.001

Polyps	2741 (34.5)	365 (34.7)	ns

Inflammation	1249 (15.7)	69 (6.9)	<0.001

Cancer	916 (11.5)	48 (4.5)	<0.001

Angiodysplasia/varices	172 (2.2)	37 (3.5)	0.008

## Discussion

Symptoms of haemorrhoids are also associated with other abnormalities [2]. It is feasible to exclude these before concluding that haemorrhoids are responsible for the presenting complaints. Colonoscopy is recommended [3]. However, opinions still differ on the subject whether or not to do colonoscopy in cases of complaints possibly due to haemorrhoids. It is stated that a full colonoscopy is necessary in all patients, regardless the age [4]. While others say that it might be a better option to perform an ano-rectal examination, to look for an active bleeding source [5]. Moreover, the authors of yet another study concluded that if haemorrhoids, fissures or polyps were identified as the probable site and cause of bleeding, colonoscopy and other investigations were not necessary, unless alarm-signs were present [6]. In common practice, the age of 50 years holds the threshold for colonoscopy since the incidence of colorectal cancer, diverticulosis and polyps increases as people are getting older [7].

The present study is an extension and confirmation of a previous paper on coincidental abnormalities in colon and rectum in patients with haemorrhoids [8]. The results show that above the age of 50 years it is more likely to find abnormalities besides haemorrhoids. Furthermore, these results can help the clinician to decide whether or not to do an colonoscopy.

A point of criticism is that the reason for doing the colonoscopy was not exclusively linked to complaints due to haemorrhoids. The population was not only studied because of bleeding but also for many others reasons, like abdominal complaints, changes in defecation pattern, family history of colorectal cancer, anemia and so on. The present study represents patients in secondary care undergoing endoscopy. Obviously all patients were sent by internists, gastroenterologists, and sometimes surgeons. There is no reason to think that the yield of endoscopy would be different in patients primarily studied because of haemorrhoids.

Another point of criticism could be that flexible endoscopy is not the most suitable way of detecting haemorrhoids. However, before starting the procedure peri-anal inspection is done and while retrieving the endoscope the anal canal is inspected as well [9-11].

In conclusion, this study supports the clinical approach as stated by Trilling et a. [6]. It is important to inspect the rectum and colon because significant findings proximal to the anal canal can be present in patients with haemorrhoids. Especially in older patients the doctor should be aware of this. In normal daily practice, especially bright rectal bleeding without further abdominal complaints will mostly be due to haemorrhoids in patients below the age of fifty. In case no other symptoms are present, haemorrhoids are seen during rectal examination, and there is no family history of colorectal cancer, the clinician could refrain from total colonoscopy in these patients. In patients above the age of fifty years endoscopic evaluation of the rectum and colon is advocated.

## Conclusion

Coincidental findings in the colon and rectum of patients with haemorrhoids are often present, especially in older patients. These may have important consequences, hence patients presenting with haemorrhoids should undergo full examination of the colon.

## Competing interests

The authors declare that they have no competing interests.

## Authors' contributions

MK did the searches in the dataset, did the statistics and the figure. RL had the initiated the study, is responsible for the entire dataset, and most of the writing.

Both authors read and approved the final manuscript.

## Pre-publication history

The pre-publication history for this paper can be accessed here:

http://www.biomedcentral.com/1471-230X/10/74/prepub
